# Performance of four modern whole genome amplification methods for copy number variant detection in single cells

**DOI:** 10.1038/s41598-017-03711-y

**Published:** 2017-06-13

**Authors:** Lieselot Deleye, Laurentijn Tilleman, Ann-Sophie Vander Plaetsen, Senne Cornelis, Dieter Deforce, Filip Van Nieuwerburgh

**Affiliations:** Laboratory of Pharmaceutical Biotechnology, Ghent University, Ottergemsesteenweg 460, 9000 Ghent, Belgium

## Abstract

Whole genome amplification (WGA) has become an invaluable tool to perform copy number variation (CNV) detection in single, or a limited number of cells. Unfortunately, current WGA methods introduce representation bias that limits the detection of small CNVs. New WGA methods have been introduced that might have the potential to reduce this bias. We compared the performance of PicoPLEX DNA-Seq (Picoseq), DOPlify, REPLI-g and Ampli-1 WGA for aneuploidy screening and copy number analysis using shallow whole genome massively parallel sequencing (MPS), starting from single or a limited number of cells. Although the four WGA methods perform differently, they are all suited for this application.

## Introduction

Whole genome amplification (WGA) has become an invaluable tool to perform massively parallel sequencing (MPS) in applications where only a limited number of cells is available. Well-known examples of such applications are: preimplantation genetic diagnosis (PGD), cell-based non-invasive prenatal testing (NIPT)^1^ and liquid biopsy of circulating tumor cells (CTCs)^2–4^. Several WGA methods exist to amplify DNA extracted from a limited number of cells, yielding the necessary amount of DNA required to perform MPS^5, 6^. The different WGA methods each have their advantages and disadvantages in terms of genome coverage, representation bias, error rates, yield and robustness.

The best WGA method per se does not exist, because the downstream application is also important to determine the ideal method^5^. Some WGA methods have already been compared for specific applications^5, 7–11^. Multiple displacement amplification (MDA) methods are better suited for single nucleotide polymorphism (SNP) detection while PCR-based methods are the better option for copy number variant (CNV) detection^5^. MDA methods use the high-fidelity phi29 polymerase, reducing nucleotide errors in the amplified sequences, while PCR-based methods tend to give a more uniform amplification across the genome. For the detection of CNVs, SurePlex WGA has proven its efficiency in clinical settings such as PGD^9, 12, 13^. However, results show that the WGA representation bias is still a limiting factor hampering higher resolution copy number profiles when starting from a single or a limited number of cells^9^. Fortunately, new WGA methods are being introduced that might have the potential to reduce this bias. In this study, we compared four different commercially available WGA methods for their suitability to detect CNVs after MPS.

A first method, REPLI-g single cell WGA, is a well-established MDA method^5, 14^. In a study comparing 5 different WGA methods, REPLI-g had the lowest false positive rate and was well-suited for detection of CNVs and single nucleotide variations (SNVs)^14^. This two-step protocol includes an amplification step of 8 h, but the hands-on time is short.

A second method, Ampli-1 WGA (Silicon Biosystems, Castel Maggiore, Italy), has already proven its efficiency for CNV and STR analysis in prenatal diagnosis^1^. Ampli-1 is based on a ligation-mediated PCR following a site-specific DNA digestion. Usage of non-random primers is one of the factors leading to a more homologous coverage. Unfortunately, the protocol consists of many different steps and is time consuming.

PicoPLEX DNA-Seq (Picoseq) (Rubicon Genomics Inc., MI 48108, USA) and DOPlify WGA (Reproductive Health Science, Thebarton, Australia) are the two most recently developed methods. Picoseq is a method based on the PicoPLEX/SurePlex WGA technology (Rubicon Genomics Inc., MI 48108, USA/BlueGnome Ltd., Mill Court, Great Shelford, Cambridge, UK) and does not only amplify the DNA of a sample but also results in an Illumina sequencing library for that sample. The amplification part is similar to PicoPLEX WGA, but during the second amplification step, Illumina adapters and barcodes are introduced. WGA and library preparation are performed in a single tube, decreasing possible handling errors and contamination, turnaround time and costs. The second new method, DOPlify WGA, uses an advanced Degenerate Oligonucleotide Primed PCR (DOP-PCR). The classical DOP-PCR does already exist for many years and was one of the first existing WGA technologies^15^. It has two basic principles: degenerate base-pairing primers and a slowly increasing annealing temperature during PCR. The classical DOP-PCR uses Taq polymerase with a high error rate, and uses primers that lead to an incomplete coverage^16^. An advanced DOP-PCR, such as DOPlify might circumvent the disadvantages of previous DOP-PCR by using new polymerases or primers and might thereby out-perform other recent WGA technologies. DOPlify is a short and easy two-step protocol of only 3 hours.

The objective of this study was to compare the performance of Ampli-1, REPLI-g, Picoseq and DOPlify WGA in a shallow whole genome MPS workflow for CNV detection, starting from a limited number of cells. Samples of 1, 3 or 5 cells were collected from the Loucy cell line in triplicate using micromanipulation. The samples were prepared using 4 different WGA methods followed by Illumina PCR-free MPS library preparation. The variability of the read distribution over the genome and the CNV detection performance were compared.

## Material and Methods

### Experimental design

**Figure 1:**
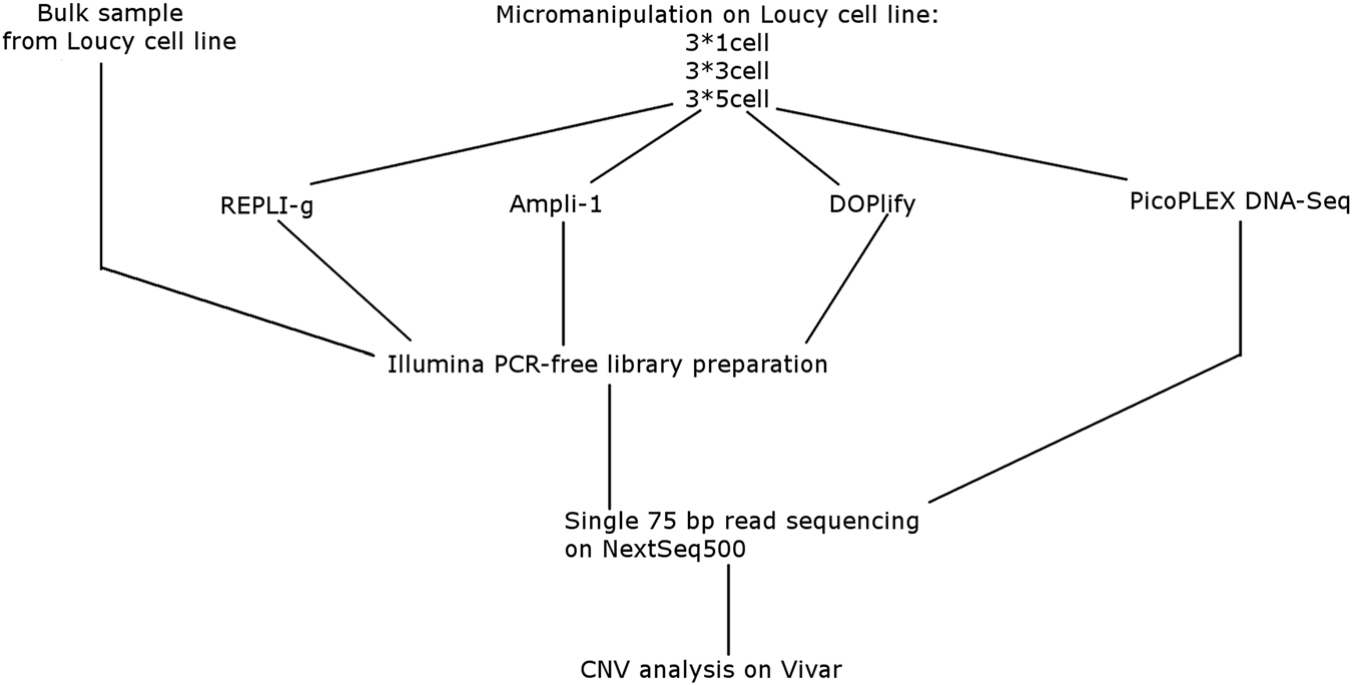
Experimental design. Samples consisting of 1, 3 or 5 cells were collected from the Loucy cell line using micromanipulation for each WGA method in triplicate. Cells were amplified with either Ampli-1, REPLI-g or DOPlify, followed by PCR-free Illumina library preparation and sequencing. A fourth method, Picoseq, performs WGA and library preparation simultaneously, without the need for a separate library preparation. A bulk DNA sample was extracted from 5 * 10^6^ Loucy cells using a column-based extraction method from Qiagen, also followed by PCR-free Illumina library preparation and sequencing.

Figure 1 illustrates the experimental design of this study. Experiments were performed on cells from the lymphoblastoid Loucy cell line (DSMZ, ACC394)^17^. A CNV analysis has been performed on this cell line as part of the COSMIC project^18, 19^. A reference 180 K arrayCGH profile (Agilent Technologies) from unamplified DNA of a bulk sample of the cell line was available from a previous study^7^ (Supplementary Figure 1). The following reference aneuploidies and CNVs (≥3 Mb) were present: a deletion of an entire X-chromosome, two deletions of ±45 Mb (consists of 6 Mb and a 36.5 Mb deletion interspersed by a 2.5 Mb normal ploidy region) and 30 Mb on respectively 5q14.3-q31.1 and 5q33.1-q35.3, a deletion of ±60 Mb on 6q21-q27, a deletion of 3 Mb on 12p13.31-p13.2, a ±26 Mb duplication of 13q31.3-q34, and two deletions of 16 Mb and 3 Mb on respectively 16p13.3-p13.11 and 16q24.2q24.3. The COSMIC and 180 K arrayCGH CNV analyses are completely concordant regarding these >3 Mb insertions and deletions.

The cell line was grown in suspension, which allows collection of individual cells. Samples containing 1, 3 or 5 cells were collected in triplicate using micromanipulation. These samples were used to perform WGA, PCR-free Illumina library preparation and sequencing. This was performed in parallel for three types of WGA methods. A fourth method, Picoseq, performs WGA and library preparation simultaneously, without the need for the PCR-free Illumina library prep. A bulk DNA sample of the Loucy cell line was also included in the study. The bulk DNA was treated in same way as the other samples, except for the omission of the amplification step.

A negative control, processed alongside the cell-containing samples, was used during all WGA methods to control for contamination. These negative controls contained the exact same reagents as the cell-containing samples except that 1 µl PBS instead of cells was added. A sample containing 1 µl of male control DNA (Human Genomic DNA, Roche, stock solution of 0.2 mg/ml) with a concentration of 30 pg/µl was used as a positive control during each WGA method. This positive control was performed to check the yield of the WGA procedure on a sample with high quality input DNA, excluding possible suboptimal conditions due to the cell manipulation and extraction steps. This sample could be used as a troubleshooting tool in case the cell samples would yield no (or low) amounts of WGA product.

The different WGA methods were compared to assess their suitability for downstream CNV analysis using shallow whole genome MPS (see other Material and Methods sections for details): A comparison of the DNA yield after WGA, the mappability of the reads and the variance in read counts per window across the genome was performed within and between the WGA samples. The CNV calling accuracy after WGA was evaluated by comparing the CNV calls in WGA samples with those called in the bulk DNA sample. As the WGA samples and the bulk sample are processed and analyzed entirely the same way except for the WGA step, this allows a comparison in which the effect of the WGA on CNV calling accuracy/resolution can be studied. The resolution is configurable and should be set considering the extent of representation bias and considering the average sequencing depth. A lower resolution, meaning that smaller CNVs will not be detected, allows for a better averaging/smoothing of representation bias introduced by the WGA and sequencing, avoiding incorrect CNV calls. We have shown before that the representation bias introduced by current WGA and sequencing methods is the limiting factor that is necessitating a resolution of ~3 Mb^7, 9^. For this reason, we compare the CNV calls in WGA samples with those called in the bulk DNA sample at a resolution of 3Mb.

### Growth and isolation of cells

The cells were grown in Rose Roswell Park Memorial Institute (RPMI-1640) medium (Life Technologies, Carlsbad, USA), supplemented with 10% fetal bovine serum (Life Technologies, Carlsbad, USA) at a temperature of 37 °C and a 5% CO_2_ level. A known number of cells was collected with a denuding handle from STRIPPER (Origio, Måløv, Denmark) and MXL3-100 needles with a diameter of 100 µm (Origio, Måløv, Denmark) from a serial dilution of medium with phosphate buffered saline (PBS). All cells were collected in a maximum total volume of 2.5 µl and snap frozen in liquid N_2_ immediately after collection. For the bulk DNA sample, DNA was extracted from 5 * 10^6^ cells using the DNeasy Blood & Tissue kit (Qiagen Hilden, Germany).

### Ampli-1 WGA, REPLI-g WGA, DOPlify WGA

Cell lysis and amplification was performed, using the Ampli-1 WGA kit (Silicon Biosystems, Castel Maggiore, Italy), the REPLI-g single cell kit (Qiagen Hilden, Germany) or the DOPlify WGA kit (Reproductive Health Science, Thebarton, Australia) as described in the respective manufacturer’s instructions. All samples were purified following the manufacturer’s protocol of the Genomic DNA Clean & Concentrator kit (version 1.0.0, Zymo Research, Irvine, USA) with 5X binding buffer. Concentration was measured using the Qubit dsDNA High Sensitivity Assay kit (Life technologies, Carlsbad, USA).

### PicoPLEX DNA-Seq

Cell lysis, amplification and library preparation was performed, using the PicoPLEX DNA-Seq kit (Rubicon Genomics Inc., MI 48108, USA) as stated in the manufacturer’s instructions. All samples were purified with 50 µl of Agencourt AMPure XP beads. The quality of the libraries was assessed with the Agilent High-Sensitivity DNA kit (Bioanalyser, Agilent Technologies, California, USA). This method will further be referred to as Picoseq.

### TruSeq DNA PCR-free HT library preparation

The WGA products from Ampli-1, REPLI-g, DOPlify and the bulk sample were fragmented to an average size of 350 bp using the S2 Focused Ultrasonicator (Covaris, Woburn, USA) following the manufacturer’s instructions (Duty cycle of 10%, Intensity of 5 and 200 cycles/burst, Duration 45 sec). Between 350ng and 1 µg of WGA product was used as input for fragmentation. Sequencing libraries of the fragmented samples were prepared with the TruSeq DNA PCR-free HT library preparation kit (Illumina) on the IP-Star Compact (Diagenode, Seraing, Belgium). Library quantification was performed using a Sequencing Library qPCR Quantification kit (Illumina, San Diego, USA) to quantify the sequenceable DNA fragments containing the correct adapters (this was performed on the samples of all four methods). The libraries from the different samples were pooled equimolarly, denatured and diluted to a final loading concentration of 2.5pM for sequencing. Finally, single-end indexed 75 bp sequencing was performed on a high-output NextSeq500 flow-cell (Illumina, California, USA).

### Data analysis

Fastq files of the samples were automatically analyzed using the Vivar software^20^. The Vivar software maps the reads using bowtie2^21^ with–verysensitive-local setting and performs CNV detection using the QDNAseq algorithm^22^. This algorithm divides the genome in equally sized windows (configurable; see below). The reads that map in these windows are counted and normalized for bias in GC-content and mappability. In addition, QDNAseq excludes anomalous genetic regions from the analysis making use of a “blacklist” based on information from the ENCODE Project Consortium^23^. The blacklist contains chromosomal regions with known repeat elements, such as satellites, centromeres, and telomeres. After GC-content and mappability normalization, read counts were median-normalized by dividing the number of reads in each window by the median number of reads across all windows. As chromosomal aberrations were assumed rare, the median number of reads across all windows is a fair estimate of the expected number of reads per window for a perfectly diploid genome. As such, the median-normalized read counts represent a measure for the deviation from diploidy for each window and a copy number (CN) estimate was calculated using following formula: CN = 2(read count per window/median read count per window). Then, a circular binary segmentation (CBS) algorithm was applied which detects breakpoints between the windows, and groups them between breakpoints into larger contiguous regions with an equal CN. To this end, the CBS algorithm started with the whole chromosome and segments it recursively by testing for change-points between such regions. The two-sample t-statistic were then applied to compare the mean of the read counts of the windows contained in one segment to the read counts of the windows in its adjourning segment. The mean of read counts of the windows contained in the segments were used as an estimator of the copy number of the whole segment. After this segmentation, CNVs were called when the segment’s log_2_(CN/2) surpasses a threshold of +/−0.35, corresponding to a CN greater than 2.55 or less than 1.57. These thresholds were chosen based on literature review and own experience^24^. From previous experience, a window size of 1 Mb should be ideal to detect chromosomal variants of 3 Mb and bigger^9^. Typically, 3 neighboring windows with a significantly higher or lower read count are required to call a CNV. When 3 consecutive windows of 1 Mb are needed to call a CNV, the detection limit is thus 3 Mb. As the WGA methods in the current study might perform better than previously tested methods, results were also generated using 500 kb windows, possibly lowering the detection limit. Analyzed data were visualized as line plots, in which windows are ordered along the x-axis by their genomic positions, and the y-axis shows the median normalized log_2_ transformed read counts, i.e. the log_2_(CN/2). Chromosomes are identified along the x-axis by an alternating white and grey background color. Each dot on the profiles represents a different window and the horizontal lines refer to the segments. Each genomic profile was checked manually for variants. Raw sequencing data are deposited in the NCBI Sequence Read Archive under project accession number PRJNA362886. On Vivar they are available under the project: Comparing four WGAs (https://holmes.ugent.be:9090/vivar/).

### Statistical analysis of the read count variance

For each sample, the average read count variance observed between the windows across the whole genome was calculated using following formula:

$$\frac{{\sum }_{i}^{N-1}{((\frac{{x}_{i}}{a})-(\frac{{x}_{i+1}}{a}))}^{2}}{N}\,$$

where ‘*N’* is the number of windows, ‘*x*_*i*_’ the read count in window *i*, ‘*x*_*i*+*1*_’ the read count in the next window *i* + *1* and *‘a’* the median number of reads across all windows^22, 25^. In this formula, the read count in each window was scaled by factor ‘a’, normalizing the result for the total number of reads that was sequenced for the sample. This measure was calculated for each sample. A one-way ANOVA was performed between the average read count variances of the four WGA methods. P-values smaller than 0.05 were considered statistically significant.

### True and false positives

True positives are defined as the CNVs that are called in the bulk DNA aCGH result. Calls that are not present in these reference samples are considered false positives. Results of the WGA and the bulk DNA samples were compared using 1 Mb windows and using 500Kb windows in the QDNAseq analysis. Within each comparison, the same window size is used for all samples. Individual cells from the same tissue or from the same cell line can show different CNVs^26, 27^. Thus, some of the false positives and false negatives in the individual cells (or in the limited number of cells samples) could have been caused by mosaicism in the Loucy cell line.

## Results

### DNA yield after WGA and DNA fragment-size

DNA yield after WGA was compared between Ampli-1, REPLI-g and DOPlify. Yields of the 1-, 3-, and 5-cell samples don’t differ significantly per method and are reported here as an average ± standard deviation. Picoseq does not yield amplified fragments, but results in sequence-ready libraries. Therefore, only the yield of adaptor-ligated fragments and not the entire DNA yield is important for this method. Ampli-1 and DOPlify WGA resulted in a comparable yield of 19.2 ± 6.9 ng/µl and 21.1 ± 2.6 ng/µl (in 30 µl), respectively. REPLI-g WGA has a significantly higher yield of 900.7 ± 255 ng/µl (in 30 µl). All negative controls contained a negligible amount of DNA after WGA, except the one amplified with REPLI-g. The concentration of 1000 ng/µl in the latter was expected: Phi29 DNA Polymerase has an extremely high processivity and will extend primer-dimers that may be present in the reaction in the absence of specific template in the reaction, leading to unspecific high molecular weight amplification products. Samples with template yield approximately the same amount of DNA as non-template controls. For all WGA methods, the yield of the positive control sample was similar to the 5-cell samples.

The average size of the DNA fragments after amplification varies between methods. REPLI-g has the longest fragments (>10Kb), Picoseq the shortest (654 ± 31.1 bp). Ampli-1 and DOPlify result in fragments of respectively 740.1 ± 27.6 bp and 957.3 ± 87.2 bp. The WGA product is fragmented to ~350 bp by sonication prior to sequencing.

### Mapping statistics

The average sequencing depth was similar for all samples and was around 0.3X. An average depth of 0.3X exceeds the required average depth to call CNVs with a resolution of 3 Mb. It has been shown before that the average depth can be reduced to 0.03X without becoming the limiting factor for the resolution: The representation bias introduced by the WGA and sequencing is the limiting factor that is necessitating larger window sizes (and thus a lower resolution)^7, 9^. For the REPLI-g, Ampli-1, DOPlify and Picoseq amplified samples, respectively 99.7 ± 0.01%, 98.8 ± 0.2%, 98.3 ± 0.7% and 97 ± 3.1% of the reads mapped to the human reference genome.

### Variance in read counts per window across the genome

**Figure 2:**
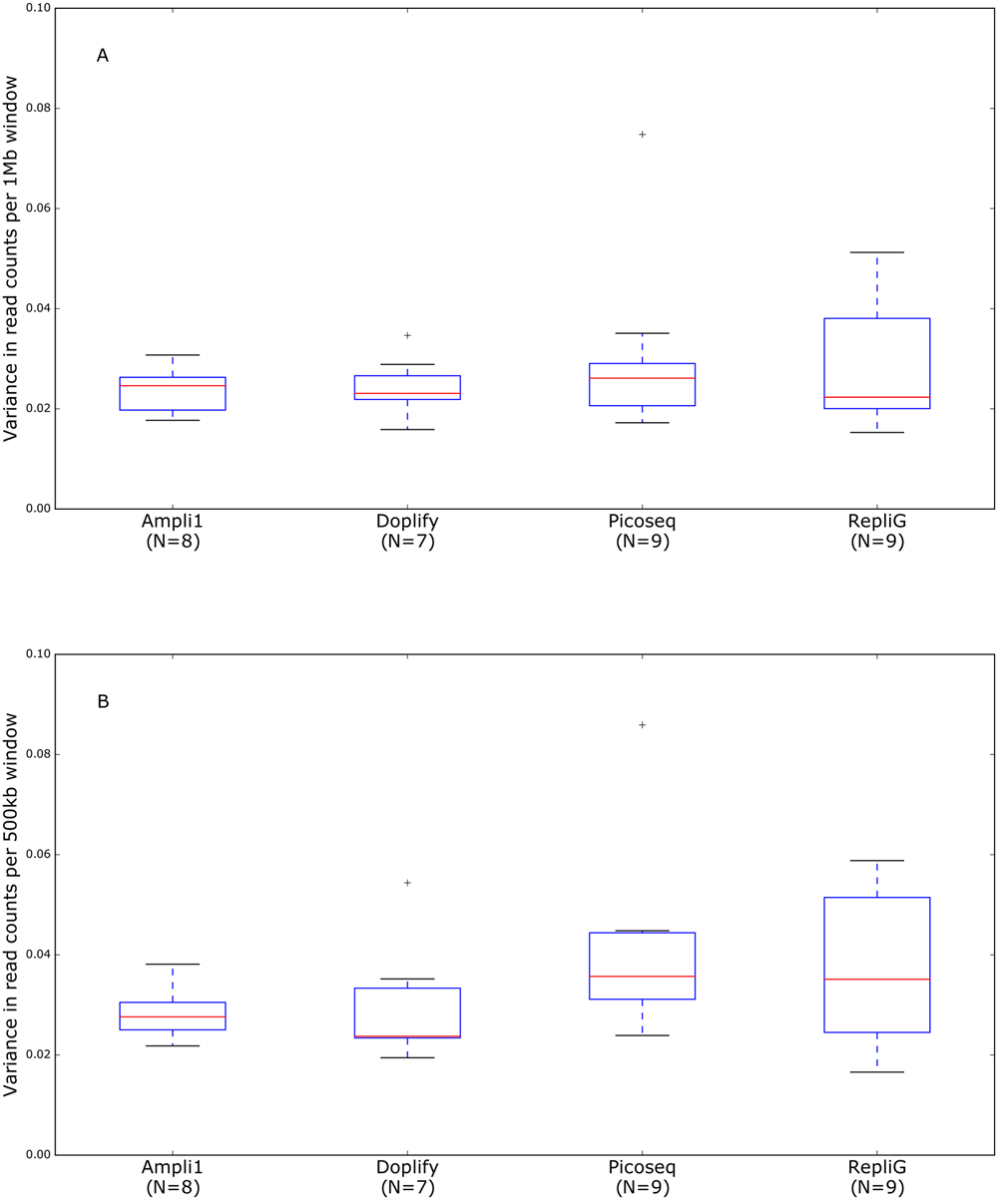
Boxplots of the average variance in read counts per window across the genome. The boxplots show all samples per WGA method. (**A**) Using 1 Mb windows. (**B**) Using 500 Kb windows. The blue box contains the values between the first and third quartiles (Q1 and Q3). The black lines are the minimum and maximum, not considering outliers. Values that are more than 1.5 times the interquartile range (Q3-Q1) beyond the closest (Q1 or Q3) quartile were considered as outliers.

**Figure 3:**
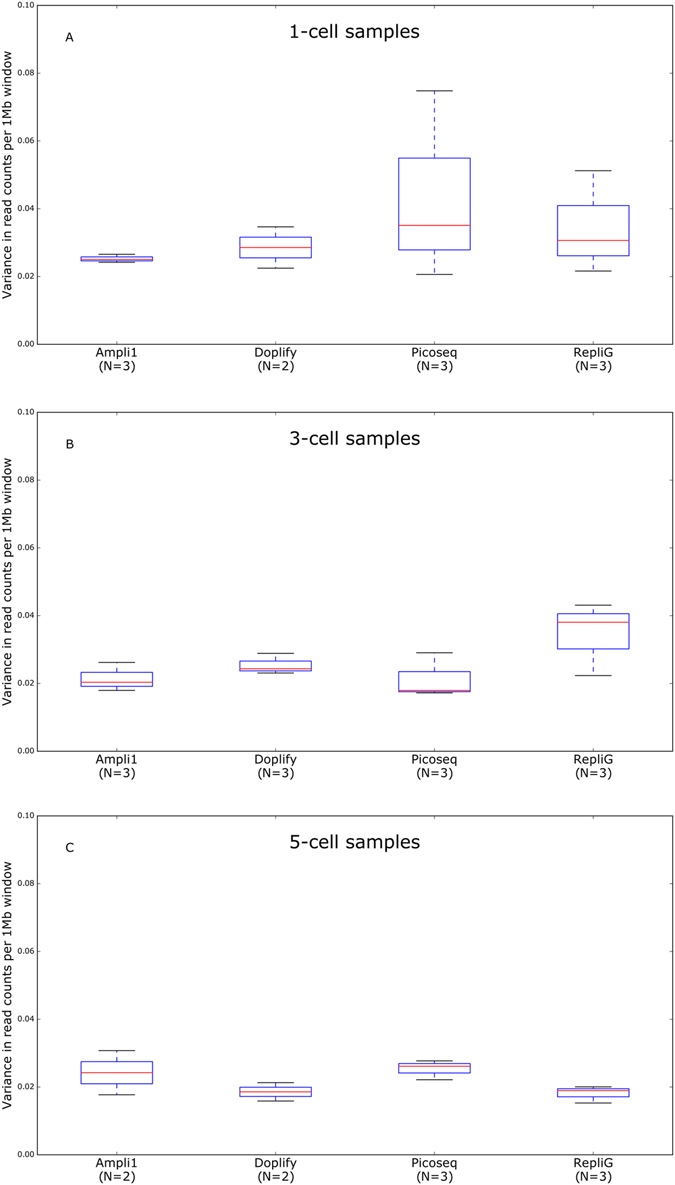
Boxplots of the variances in read counts per 1 Mb window across the genome for 1-, 3- and 5-cell samples. (**A**) All 1-cells samples per WGA method, except the failed one. (**B**) All 3-cell samples per WGA method. (**C**) All 5-cell samples per WGA method, except the failed ones. The blue box contains the values between the first and third quartiles (Q1 and Q3). The black lines are the minimum and maximum, not considering outliers. Values that are more than 1.5 times the interquartile range (Q3-Q1) beyond the closest (Q1 or Q3) quartile were considered as outliers.

A uniform distribution of the reads over the windows across the genome (except for the parts with a CNV) is needed to be able to accurately call CNVs. Almost all the samples in this study had such uniform distribution. One 5-cell sample from Ampli-1 as well as a 1-cell and 5-cell sample from DOPlify showed a distribution that cannot be used to call CNVs as the per-window read counts across the genome are so irregular that incorrect CNVs would be called across the entire genome (Supplementary Figure S2). Those three failures, as they are further referred to, were excluded from any further analyses and are not included in the statistical analyses. Overall, the average variance in read counts per window across the genome was similar for all samples amplified with the same WGA method (Fig. 2). Furthermore, this average variance was not significantly different between the four WGA methods (Fig. 2). When the 1-cell, 3-cell and 5-cell samples were compared separately between all methods, they also showed no significantly different average variance (Supplementary Table S1; Fig. 3). Although, statistically there was no difference, some observations were made. Picoseq seems to have a higher average read count variance for single cell amplification, with the highest difference in average variance between the three single cell samples amplified with this WGA (Supplementary Table 1; Fig. 3A). For all WGA methods, the 3- and 5-cell samples tend to have a slightly lower average variance compared to single cell samples (Supplementary Table S1; Fig. 3).

### CNV line profiles with 1 Mb windows

**Figure 4:**
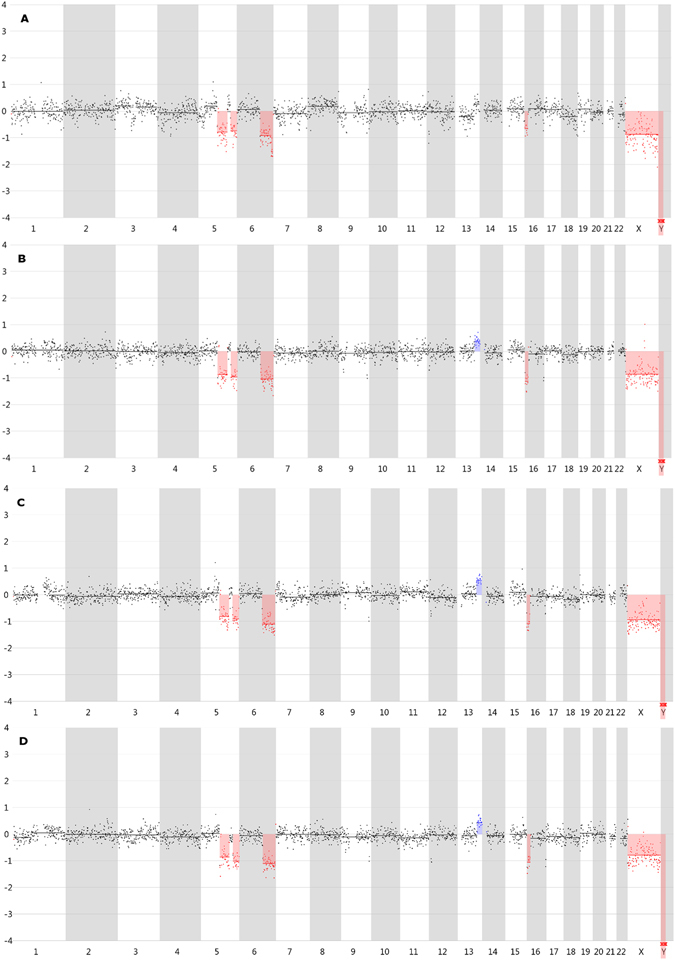
CNV line profiles generated with Vivar using 1 Mb windows. (**A**) A line profile of a 3-cell sample amplified with REPLI-g. (**B**) A line profile of an Ampli-1 amplified 3-cell sample. (**C**) A line profile of a DOPlify amplified 3-cell sample. (**D**) A line profile of a Picoseq amplified 3-cell sample. The blue color indicates a duplication or trisomy, whereas the red color indicates a deletion or monosomy.

**Figure 5:**
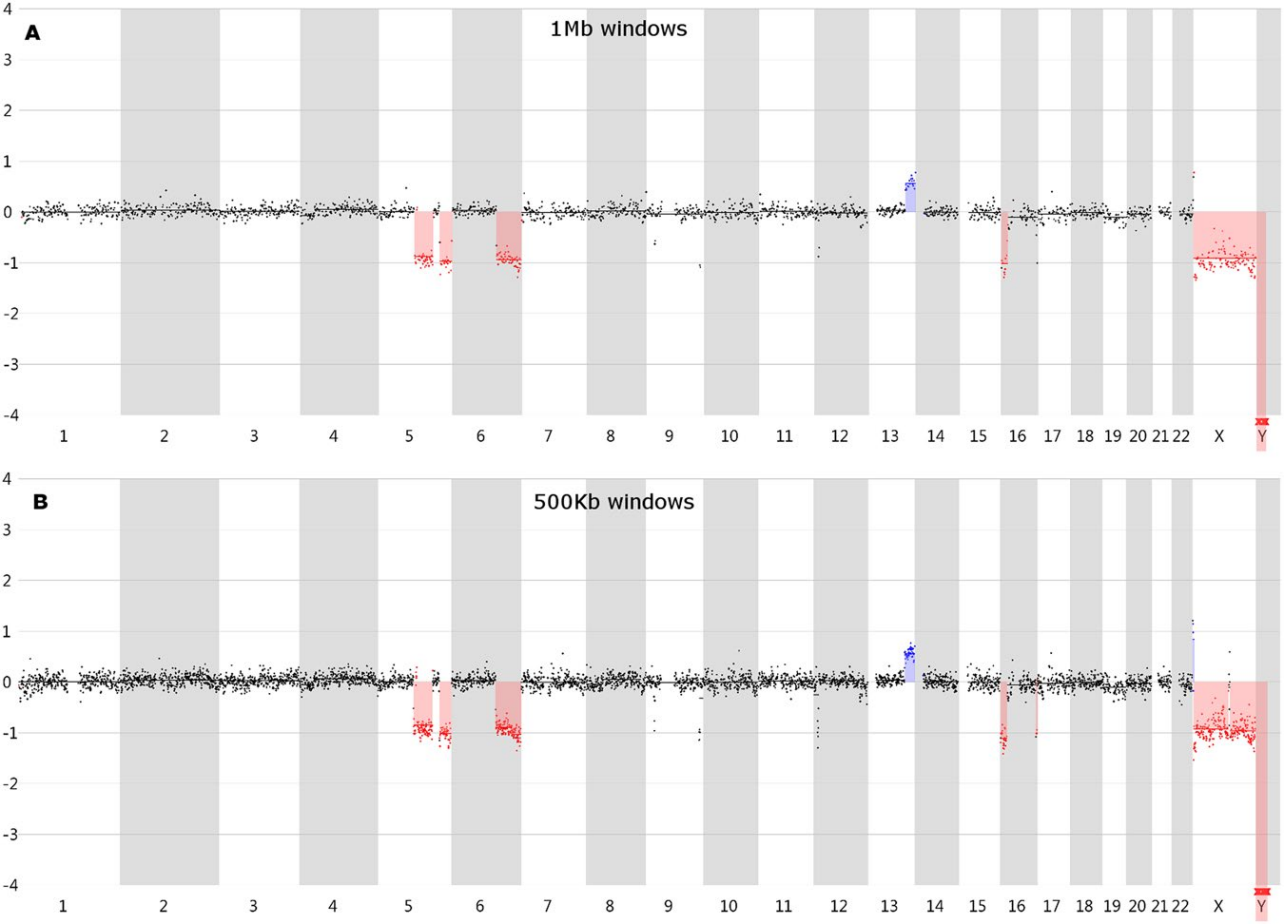
Line profile of the non-amplified bulk reference sample. (**A**) The line profile using 1 Mb windows. (**B**) The line profile using 500 Kb windows. The blue color indicates a duplication or trisomy, whereas the red color indicates a deletion or monosomy.

The 1 Mb window line profiles of all samples are available online (https://holmes.ugent.be:9090/vivar/) and in Supplementary Figure S3. One example of a 1 Mb windows profile of a 3-cell sample is shown in Fig. 4 for each WGA method. The 1 Mb window profile of the sequenced bulk DNA reference sample (Fig. 5A) showed all reference CNVs (>3 Mb), except the 3 Mb deletion near the start of chromosome 12 and the 3 Mb deletion at the end of chromosome 16. The same CNVs were called in almost all WGA samples. Every Picoseq sample showed the same CNVs. After DOPlify WGA, two samples failed to show a usable CNV profile, as mentioned above. In the other DOPlify amplified samples, the same CNVs were called. One sample failed after Ampli-1 WGA and was excluded from further analysis. Three out of the 8 remaining Ampli-1 samples showed no call for the 26 Mb insertion on chromosome 13. Nevertheless, the line profiles of these three samples all showed a slightly increased CN at the position of the insertion. REPLI-g samples missed the insertion on chromosome 13 in 2 out of 9 samples.

Picoseq and DOPlify did not only result in the same CNVs calls as the sequenced bulk sample, but did also result in no false positive calls. Analysis with Ampli-1 and REPLI-g was slightly less accurate because some false positives were called. Only two Ampli-1 amplified samples showed false positives, in contrast to five of the REPLI-g amplified samples. Overall, no specific trend in accuracy was observed in relation with the number of amplified cells. A table with the true and false positive calls per sample is shown in Supplementary Table S2.

### 1 Mb window size versus 500 Kb window size

By analyzing the data with smaller windows, smaller CNVs could be detected. Supplementary Figure S4 shows the 500 Kb windows line profiles of all samples. The sequenced bulk reference sample showed all reference CNVs, except the 3 Mb deletion near the start of chromosome 12 when the data was analyzed with a 500 Kb window size (Fig. 5B). Compared with this result, Picoseq and DOPlify amplified samples still showed 100% concordance. Two Ampli-1 amplified samples still missed the insertion on chromosome 13. One REPLI-g sample was still missed the insertion on chromosome 13 and one other REPLI-g sample missed the 3 Mb deletion at the end of chromosome 16.

The 180 Kb arrayCGH reference shows a 3 Mb deletion near the start of chromosome 12. Although this deletion was not called in the bulk reference sample, the 500 Kb line profile (Fig. 5B) clearly shows windows with lower read counts at this position in the genome. This deletion is called in all but one of the Picoseq samples, 5 out of 7 DOPlify samples, 4 out of 9 REPLI-g samples and 4 out of 8 Ampli-1 samples. The 180 Kb arrayCGH reference shows also a 1.5 Mb Chr9p and a 2.5 Mb Chr9q deletion. Although not called in the bulk reference sample, the 500Kb windows profile (Fig. 5B) clearly shows windows with lower read counts at these positions in the genome. The 1.5 Mb Chr9p deletion is only called in one REPLI-g sample. The Chr9q deletion is called in 5 out of 9 REPLI-g samples and 2 out of 7 DOPlify samples. Finally, the arrayCGH reference shows a small insertion and a small deletion on Chr16q. The 500 Kb windows line profile of the bulk reflects this, albeit not clearly. However, this deletion is called in 4 out of 8 Ampli-1 samples and 1 out of 9 Picoseq samples.

Considering all other calls as false positives, Picoseq and DOPlify did not introduce any false positives in the 500 Kb windows analysis. Ampli-1 amplified samples showed almost 2 times as much false positives with 500 Kb windows compared to 1 Mb windows. REPLI-g even showed 3 times more false positives compared to the analysis with 1 Mb windows.

## Discussion

In this study, the performance of 4 different WGA methods was investigated to examine if they generate a WGA product from a limited number of cells that is suitable for CNV detection after shallow whole genome sequencing. All four methods are suitable for this goal, showing only minor differences in the results.

REPLI-g WGA resulted in the highest yield after amplification, enabling the use of this WGA material for multiple downstream applications. DOPlify and Ampli-1 yielded only enough DNA to perform one PCR-free library preparation. Picoseq resulted in a sequencing-ready library, using a single-tube reaction. After library preparation, only the yield of adaptor-ligated, sequenceable fragments is of essence.

All WGA methods showed a high percentage of reads mapping to the human genome. In this respect, the four WGA methods showed similar results. All methods showed a uniform distribution of reads across the genome with a similar average variance in read counts per window across the genome. Ampli-1 failed to amplify one 5-cell sample and DOPlify failed to amplify a 1-cell and a 5-cell sample. The reason for this failure is unclear. It might be that these protocols are less robust or the input material might have been of bad quality. The latter is unlikely since all cells were isolated simultaneously. The WGA products and sequencing libraries of these failed samples showed a similar size distribution as the successful samples during the quality check using an Agilent bioanalyser and could thus not be excluded before sequencing.

The accuracy to detect CNVs showed some discrepancies between the four tested methods. When using 1 Mb windows, DOPlify and Picoseq samples detected 100% of the CNVs that were also detected in the sequenced bulk sample. The sensitivity to detect CNVs was lower for Ampli-1 and REPLI-g. In some of these samples the 26 Mb insertion on chromosome 13 was not called. The size of this insertion is well above the threshold of 3 Mb which is approximately the detection limit when using 1 Mb windows. Using 1 Mb windows, both DOPlify and Picoseq had a CNV profile with only the expected CNVs and without false positives. A few false positives were detected in Ampli-1 amplified samples and even more were detected in REPLI-g amplified samples. Although this is not obvious from the line profiles, nor the average variance in read counts in windows across the genome, the (local) CNV signal-to-noise ratio seems to be lower in the Ampli-1 and REPLI-g amplified samples. The metric of the average variance in reads counts in windows across the whole genome is indeed not a good metric to show possible variance in specific smaller regions of the genome.

In an effort to increase the resolution and to detect smaller CNVs, an analysis using 500Kb windows was performed. Also in this analysis Picoseq and DOPlify excelled, leading to the highest number of detected true positives without detection of false positives. While additional true positives were also detected in the Ampli-1 and REPLI-g amplified samples, the number of false positives increased substantially in the high-resolution analysis.

## Electronic supplementary material

Supplementary Info

## References

[1] Breman AM (2016). Evidence for feasibility of fetal trophoblastic cell-based noninvasive prenatal testing. Prenat. Diagn..

[2] Lohr JG (2014). Whole-exome sequencing of circulating tumor cells provides a window into metastatic prostate cancer. Nat. Biotechnol..

[3] Polzer B (2014). Molecular profiling of single circulating tumor cells with diagnostic intention. EMBO Mol Med..

[4] Zhang C, Guan Y, Sun Y, Ai D, Guo Q (2016). Tumor heterogeneity and circulating tumor cells. Cancer Lett..

[5] De Bourcy CFA (2014). A quantitative comparison of single-cell whole genome amplification methods. PLoS One.

[6] Macaulay, I. C. & Voet, T. Single Cell Genomics: Advances and Future Perspectives. *PLoS Genet*. **10** (2014). 10.1371/journal.pgen.1004126 PMC3907301 24497842

[7] Deleye L (2015). Whole genome amplification with SurePlex results in better copy number alteration detection using sequencing data compared to the MALBAC method. Sci. Rep..

[8] Deleye L (2016). Performance of a TthPrimPol-based whole genome amplification kit for copy number alteration detection using massively parallel sequencing. Sci. Rep.

[9] Deleye L (2015). Shallow whole genome sequencing is well suited for the detection of chromosomal aberrations in human blastocysts. Fertil. Steril..

[10] Hou Y (2015). Comparison of variations detection between whole-genome amplification methods used in single-cell resequencing. Gigascience..

[11] Li N (2015). The Performance of Whole Genome Amplification Methods and Next-Generation Sequencing for Pre-Implantation Genetic Diagnosis of Chromosomal Abnormalities. J. Genet. Genomics.

[12] Fiorentino F (2014). Development and validation of a next-generation sequencing–based protocol for 24-chromosome aneuploidy screening of embryos. Fertil. Steril..

[13] Wells D (2014). Clinical utilisation of a rapid low-pass whole genome sequencing technique for the diagnosis of aneuploidy in human embryos prior to implantation. J. Med. Genet..

[14] Huang L, Ma F, Chapman A, Lu S, Xie XS (2015). Single-Cell Whole-Genome Amplification and Sequencing: Methodology and Applications. Annu. Rev. Genomics Hum. Genet..

[15] Arneson, N., Hughes, S., Houlston, R. & Done, S. Whole-Genome Amplification by Degenerate Oligonucleotide Primed PCR (DOP-PCR). *CSH Protoc*. **2008**, pdb.prot4919 (2008). 10.1101/pdb.prot4919 21356673

[16] Dean FB (2002). Comprehensive human genome amplification using multiple displacement amplification. Proc. Natl. Acad. Sci..

[17] Ben-Bassat H, Shlomai Z, Kohn G, Prokocimer M (1990). Establishment of a human T-acute lymphoblastic leukemia cell line with a (16;20) chromosome translocation. Cancer Genet. Cytogenet..

[18] Forbes SA (2011). COSMIC: mining complete cancer genomes in the Catalogue of Somatic Mutations in Cancer. Nucleic Acids Res.

[19] Forbes SA (2010). COSMIC (the Catalogue of Somatic Mutations in Cancer): a resource to investigate acquired mutations in human cancer. Nucleic Acids Res.

[20] Sante, T. *et al*. ViVar: A comprehensive platform for the analysis and visualization of structural genomic variation. *PLoS One* **9**, (2014). 10.1371/journal.pone.0113800 PMC4264741 25503062

[21] Langmead B, Salzberg SL (2012). Fast gapped-read alignment with Bowtie 2. Nat. Methods..

[22] Scheinin I (2014). DNA copy number analysis of fresh and formalin-fixed specimens by shallow whole-genome sequencing with identification and exclusion of problematic regions in the genome assembly. Genome Res..

[23] The ENCODE Project Consortium. An integrated encyclopedia of DNA elements in the human genome, doi:10.1038/nature11247 (2012). 10.1038/nature11247 PMC3439153 22955616

[24] Olshen AB, Venkatraman ES, Lucito R, Wigler M (2004). Circular binary segmentation for the analysis of array-based DNA copy number data. Biostatistics..

[25] von Neumann J, Kent RH, Bellinson HR, Hart BI (1941). The mean square successive difference. Ann. Math. Stat..

[26] McConnell MJ (2013). Mosaic Copy Number Variation in Human Neurons. Science (80-.)..

[27] Žilina O (2015). Somatic mosaicism for copy-neutral loss of heterozygosity and DNA copy number variations in the human genome. BMC Genomics..

