# Supplementary material for: Performance of four modern whole genome amplification methods for copy number variant detection in single cells
Source: Sci Rep. 2017 Jun 13;7:3422. doi: 10.1038/s41598-017-03711-y (PMC5469777; doi:10.1038/s41598-017-03711-y)
Supplement: Supplementary file 1 — Supplementary Info [file 41598_2017_3711_MOESM1_ESM.pdf]

# Performance of four modern whole genome amplification methods for copy number variant detection in single cells

Lieselot Deleye[1], Laurentijn Tilleman[1], Ann-Sophie Vander Plaetsen[1], Senne Cornelis[1], Dieter Deforce[1, #,] Filip Van Nieuwerburgh[1,#,*]

[1]Laboratory of Pharmaceutical Biotechnology, Ghent University, Ottergemsesteenweg 460, 9000 Ghent, Belgium.
[*]Corresponding author (email: Filip.VanNieuwerburgh@UGent.be)
[#]These authors contributed equally.

**Contact information:**

Filip Van Nieuwerburgh
Ghent University, Laboratory of Pharmaceutical Biotechnology
Ottergemsesteenweg 460, 9000 Ghent, Belgium
email: Filip.VanNieuwerburgh@UGent.be

Supplementary Figure S1: 180K arrayCGH of bulk DNA from the Loucy cell line. This profile shows all CNVs detected in the female Loucy cell line with a resolution of 50 kb. Red bars indicate deletions and blue bars indicate insertions.

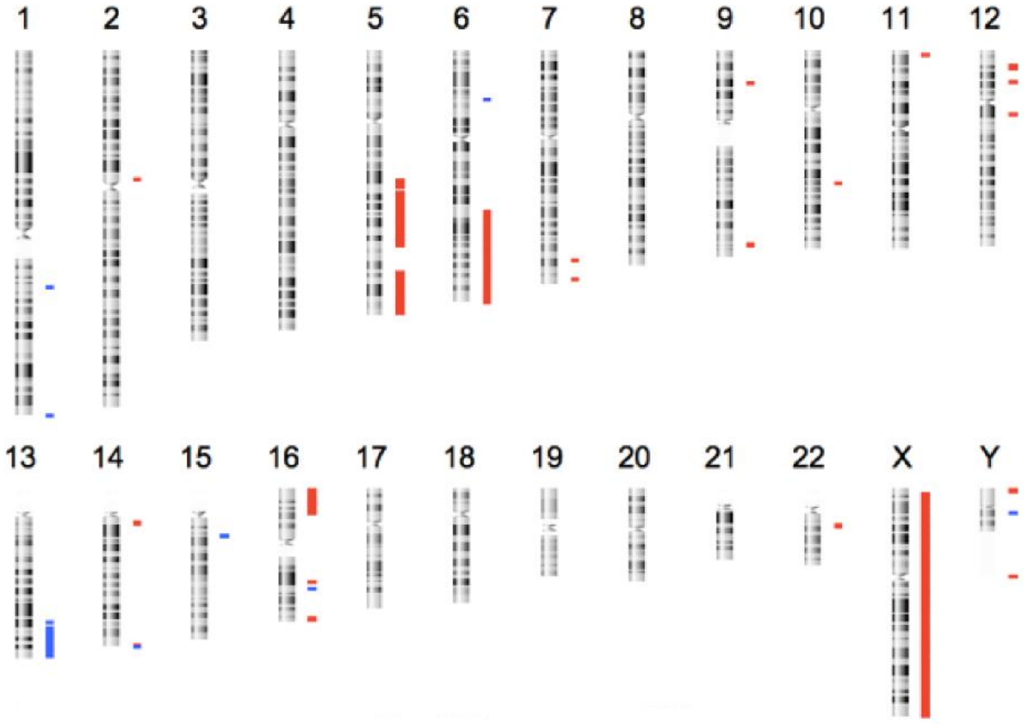

Supplementary Figure S2: Failed samples with an unusable CNV profile.

A) An Ampli-1 amplified 5-cell sample. B) & C) A DOPlify amplified 1-cell and 5-cell sample.

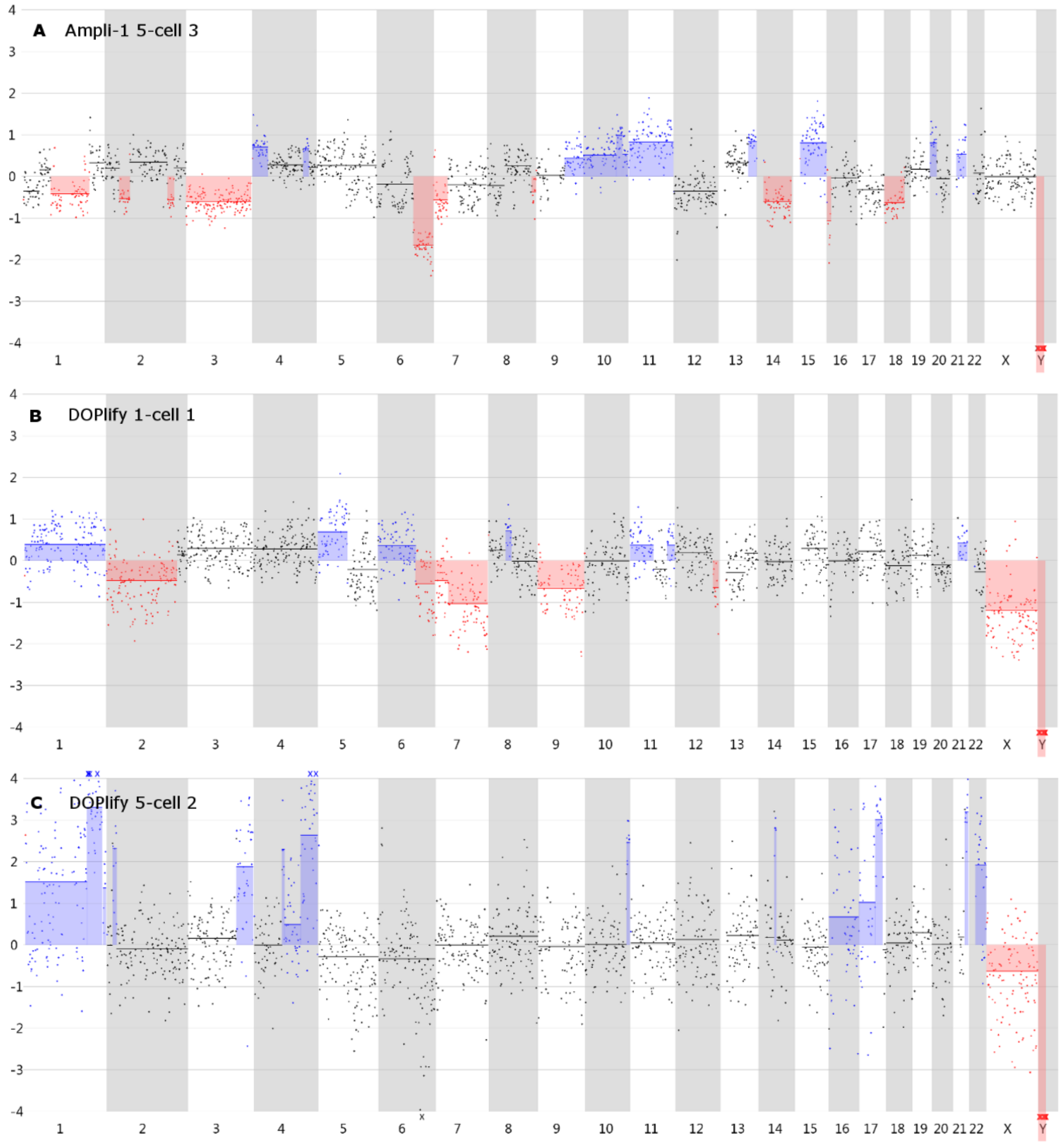

Supplementary Table S1: Average read count variances across the genome in 1Mb and 500Kb windows.

| | | 1Mb | 500Kb |
|---|---|---|---|
| Ampli-1 | 1 cell | 0.025±0.001 | 0.029±0.003 |
| | 3 cell | 0.022±0.004 | 0.026±0.002 |
| | 5 cell | 0.024±0.009 | 0.030±0.012 |
| REPLI-g | 1 cell | 0.035±0.150 | 0.034±0.015 |
| | 3 cell | 0.034±0.110 | 0.048±0.012 |
| | 5 cell | 0.018±0.002 | 0.019±0.004 |
| DOPlify | 1 cell | 0.029±0.009 | 0.045±0.014 |
| | 3 cell | 0.025±0.003 | 0.026±0.005 |
| | 5 cell | 0.019±0.004 | 0.021±0.003 |
| Picoseq | 1 cell | 0.044±0.028 | 0.054±0.029 |
| | 3 cell | 0.021±0.007 | 0.032±0.110 |
| | 5 cell | 0.025±0.003 | 0.036±0.004 |

Supplementary Figure S3: CNV line profiles of all samples for a 1Mb window. REPLI-g CNV profiles

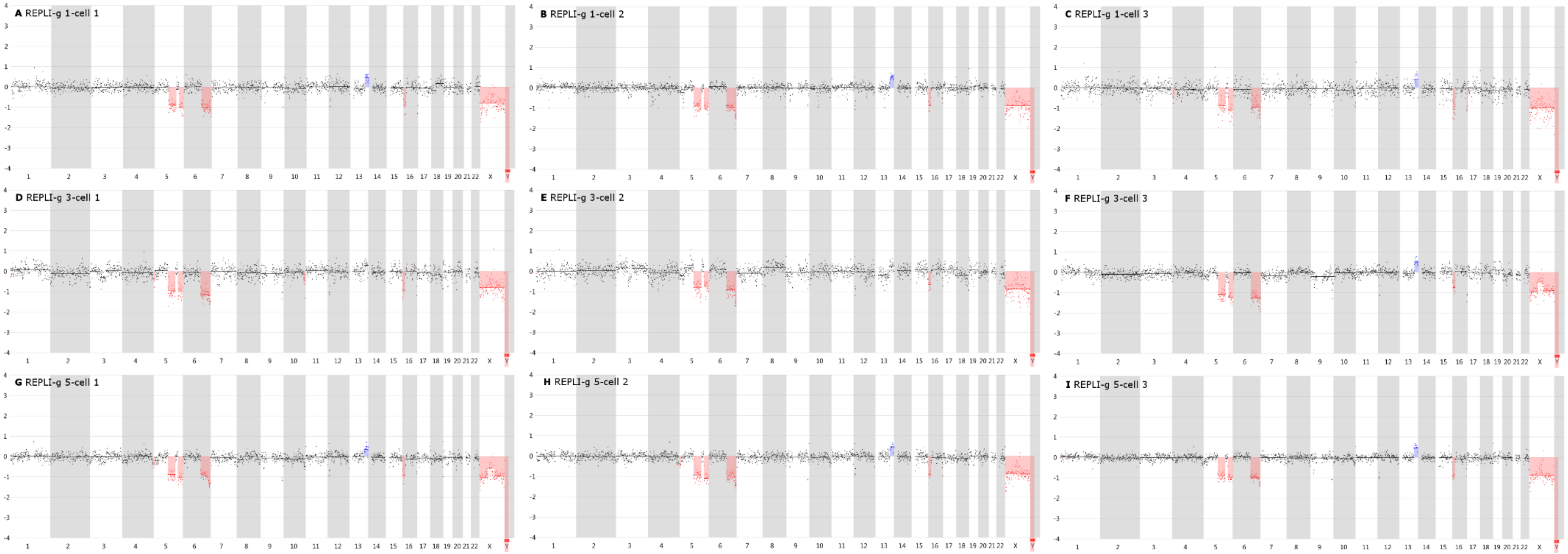

Supplementary Figure S3: CNV line profiles of all samples for a 1Mb window. Ampli-1 CNV profiles

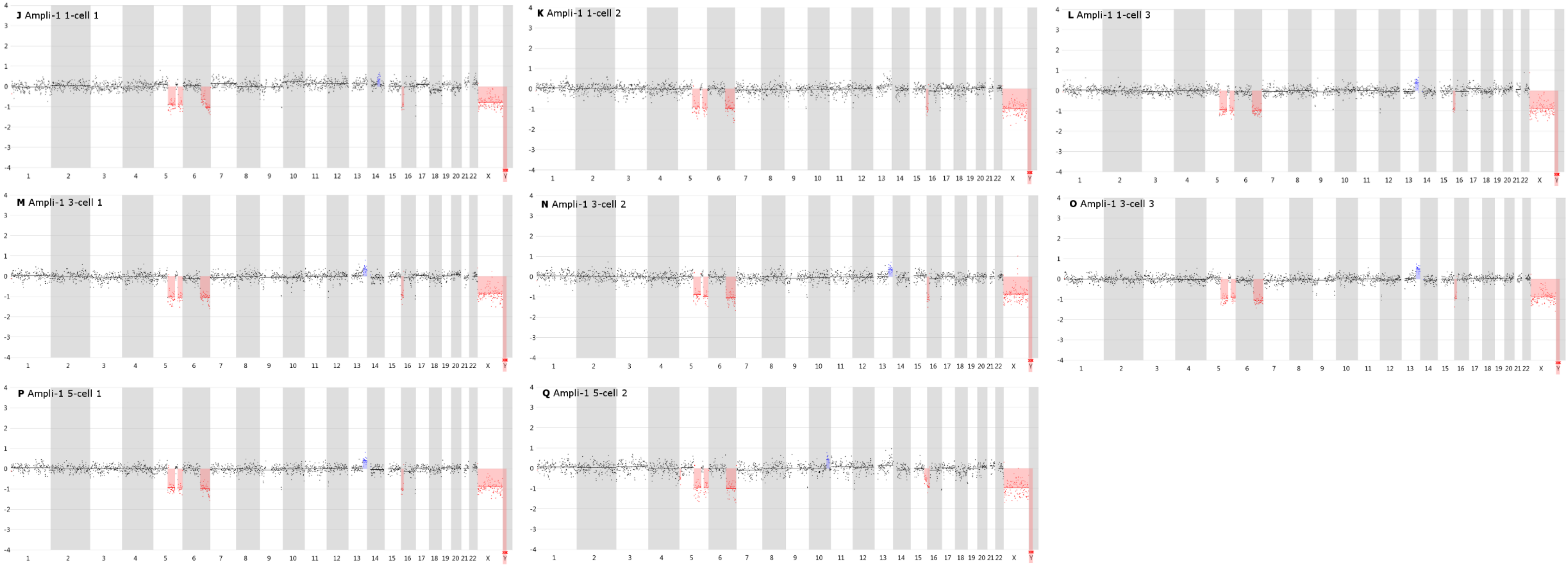

Supplementary Figure S3: CNV line profiles of all samples for a 1Mb window. DOPlify CNV profiles

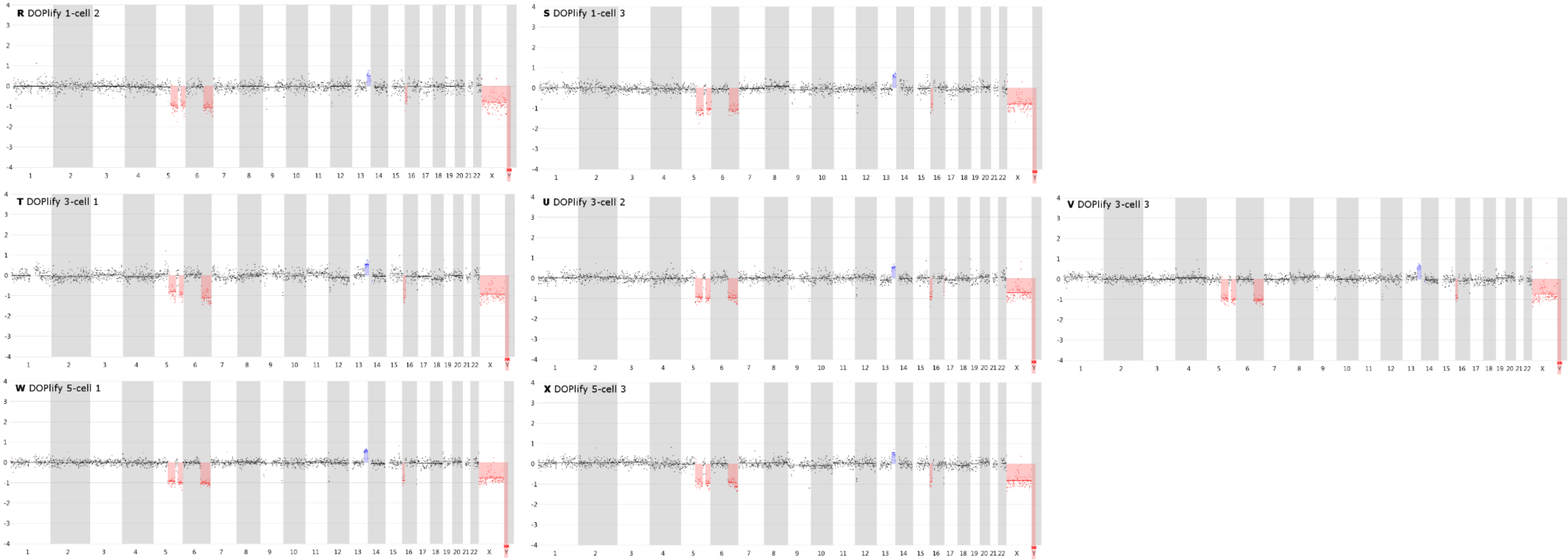

Supplementary Figure S3: CNV line profiles of all samples for a 1Mb window. Picoseq CNV profiles

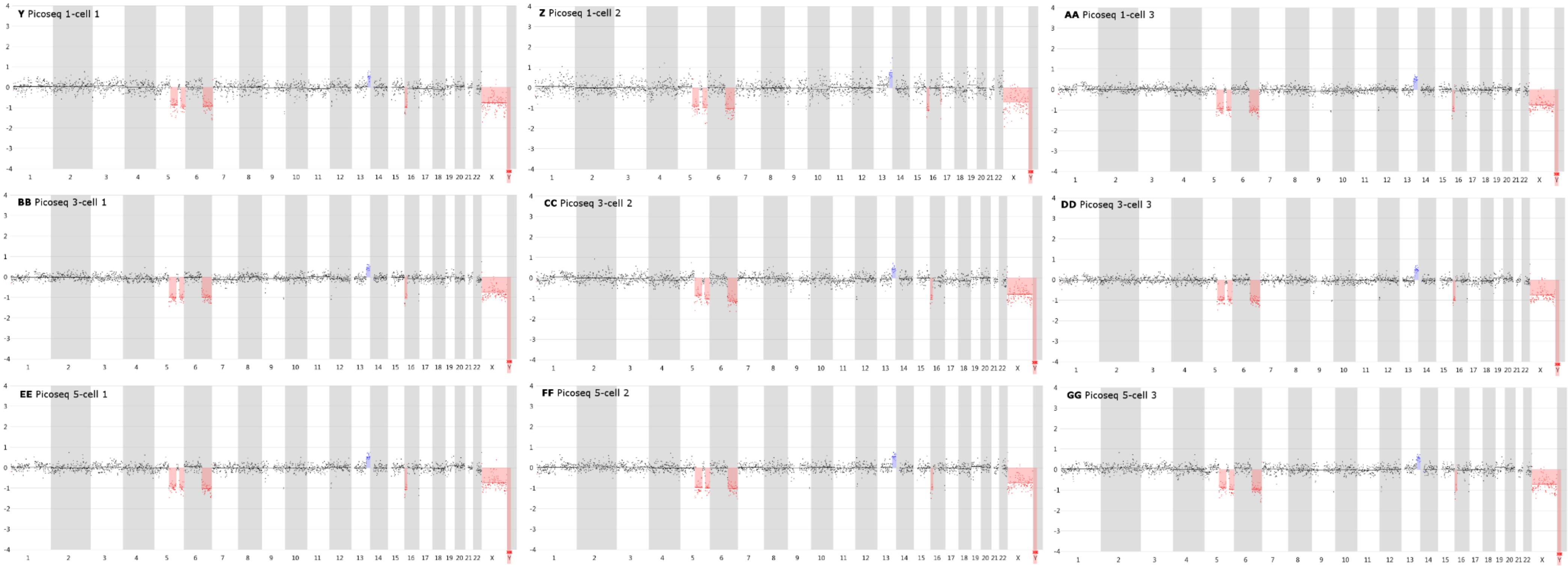

Supplementary Table S2: True and false positives called by Vivar using 1Mb windows. The left side of the table contains all the true positives that are larger than 3Mb. The right side shows the false positives. The column headers indicate the location of the insertion (**in bold**) or the deletion. Two CNVs located on the same chromosomal arm are distinguished by subscript (1) or (2).

| | | Chr5q$_{(1)}$ | Chr5q$_{(2)}$ | Chr6q | Chr12p | **Chr13q** | Chr16p | Chr16q | X-Chr | Chr4p | Chr5p | Chr8p | **Chr10q** | Chr10q | **Chr14q** | Chr15q |
|---|---|---|---|---|---|---|---|---|---|---|---|---|---|---|---|---|
| Bulk | | √ | √ | √ | | √ | √ | | √ | | | | | | | |
| Ampli-1 | 1c1 | √ | √ | √ | | | √ | | √ | | | | | | √ | |
| | 1c2 | √ | √ | √ | | | √ | | √ | | | | | | | |
| | 1c3 | √ | √ | √ | | √ | √ | | √ | | | | | | | |
| | 3c1 | √ | √ | √ | | √ | √ | | √ | | | | | | | |
| | 3c2 | √ | √ | √ | | √ | √ | | √ | | | | | | | |
| | 3c3 | √ | √ | √ | | √ | √ | | √ | | | | | | | |
| | 5c1 | √ | √ | √ | | √ | √ | | √ | | | | | | | |
| | 5c2 | √ | √ | √ | | | √ | | √ | | √ | | √ | | | √ |
| REPLI-g | 1c1 | √ | √ | √ | | √ | √ | | √ | | | √ | | | | |
| | 1c2 | √ | √ | √ | | √ | √ | | √ | | | | | | | |
| | 1c3 | √ | √ | √ | | √ | √ | | √ | √ | | | | | | |
| | 3c1 | √ | √ | √ | | | √ | | √ | | √ | | | √ | | |
| | 3c2 | √ | √ | √ | | | √ | | √ | | | | | | | |
| | 3c3 | √ | √ | √ | | √ | √ | | √ | | | | | | | |
| | 5c1 | √ | √ | √ | | √ | √ | | √ | | | | | | | |
| | 5c2 | √ | √ | √ | | √ | √ | | √ | | √ | | | | | |
| | 5c3 | √ | √ | √ | | √ | √ | | √ | | √ | | | | | |
| DOPlify | 1c2 | √ | √ | √ | | √ | √ | | √ | | | | | | | |
| | 1c3 | √ | √ | √ | | √ | √ | | √ | | | | | | | |
| | 3c1 | √ | √ | √ | | √ | √ | | √ | | | | | | | |
| | 3c2 | √ | √ | √ | | √ | √ | | √ | | | | | | | |
| | 3c3 | √ | √ | √ | | √ | √ | | √ | | | | | | | |
| | 5c1 | √ | √ | √ | | √ | √ | | √ | | | | | | | |
| | 5c3 | √ | √ | √ | | √ | √ | | √ | | | | | | | |
| Picoseq | 1c1 | √ | √ | √ | | √ | √ | | √ | | | | | | | |
| | 1c2 | √ | √ | √ | | √ | √ | | √ | | | | | | | |
| | 1c3 | √ | √ | √ | | √ | √ | | √ | | | | | | | |
| | 3c1 | √ | √ | √ | | √ | √ | | √ | | | | | | | |
| | 3c2 | √ | √ | √ | | √ | √ | | √ | | | | | | | |
| | 3c3 | √ | √ | √ | | √ | √ | | √ | | | | | | | |
| | 5c1 | √ | √ | √ | | √ | √ | | √ | | | | | | | |
| | 5c2 | √ | √ | √ | | √ | √ | | √ | | | | | | | |
| | 5c3 | √ | √ | √ | | √ | √ | | √ | | | | | | | |

Supplementary Figure S4: CNV line profiles of all samples for a 500Kb window. REPLI-g CNV profiles

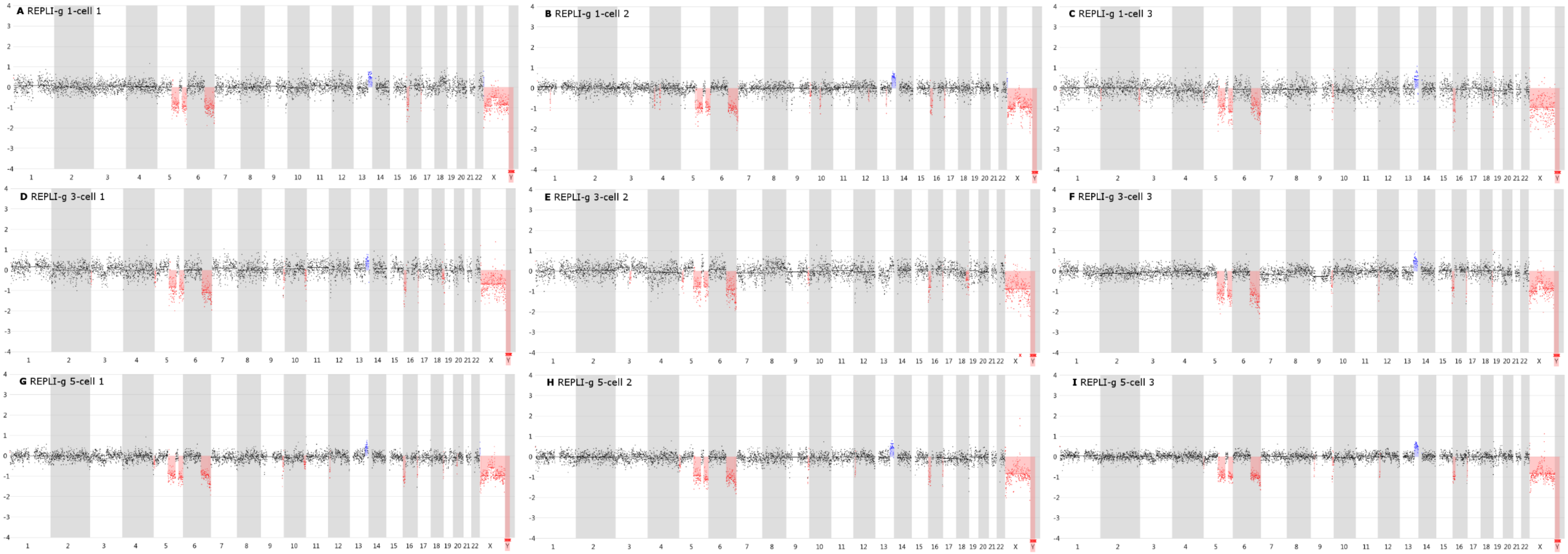

Supplementary Figure S4: CNV line profiles of all samples for a 500Kb window. Ampli-1 CNV profiles

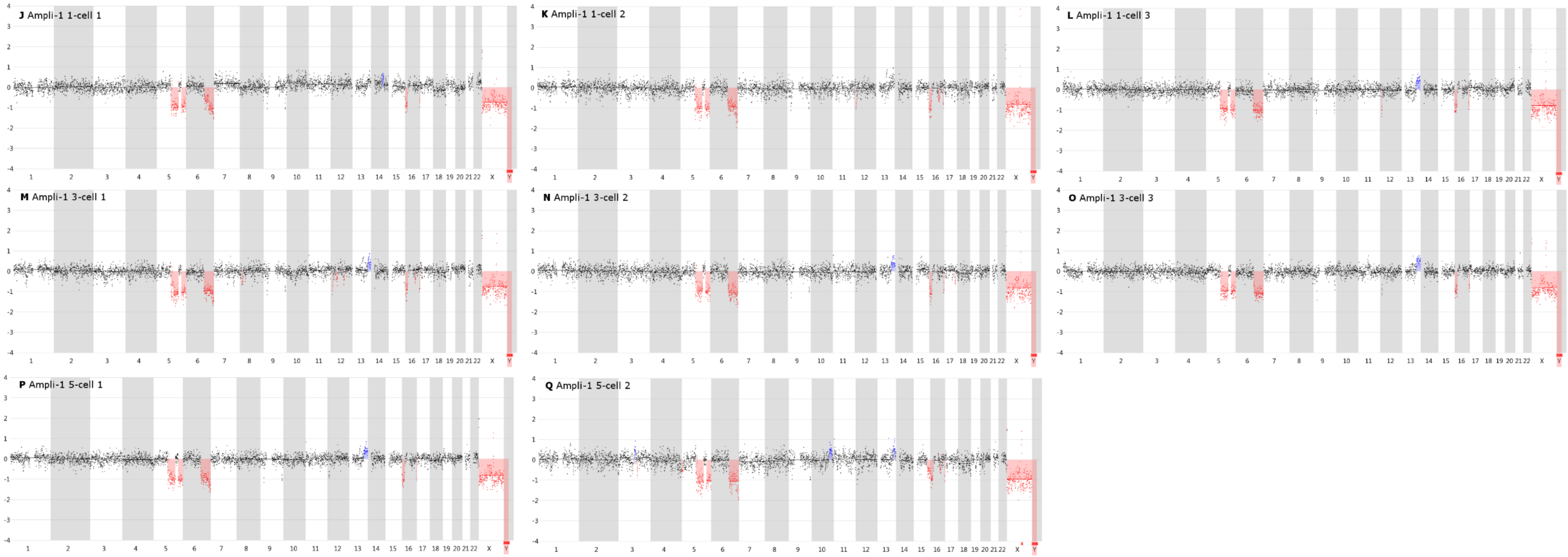

Supplementary Figure S4: CNV line profiles of all samples for a 500Kb window. DOPlify CNV profiles

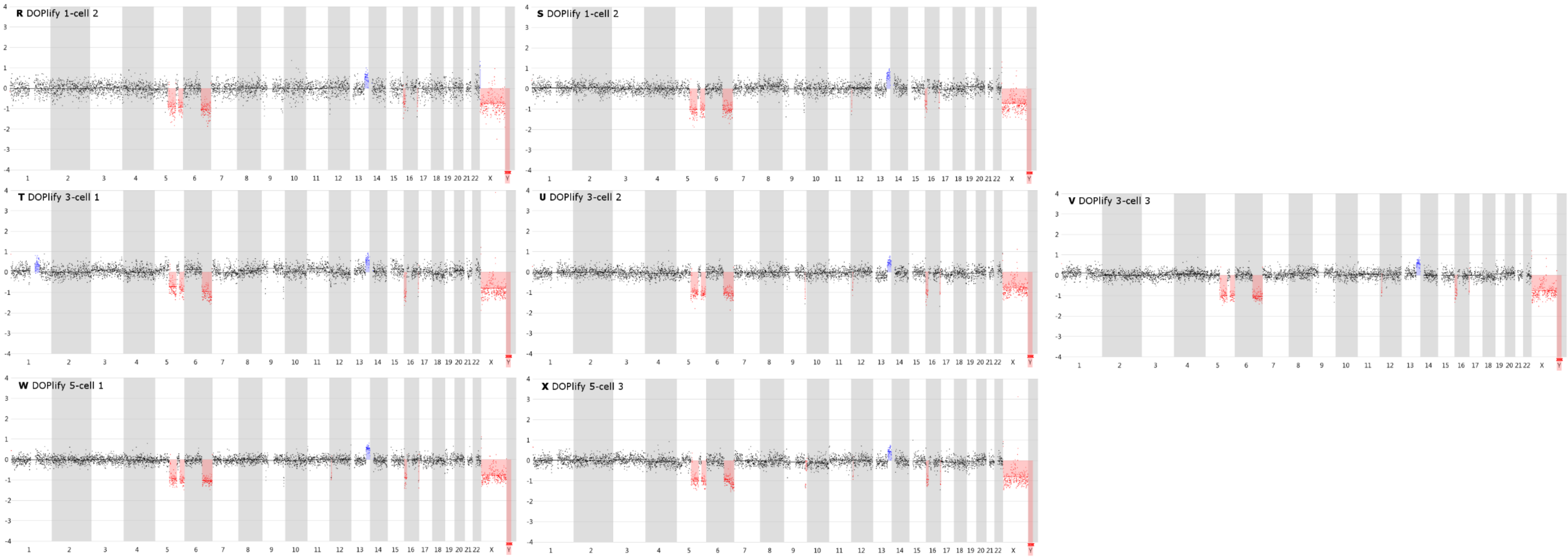

Supplementary figure S4: CNV line profiles of all samples for a 500Kb window. Picoseq CNV profiles

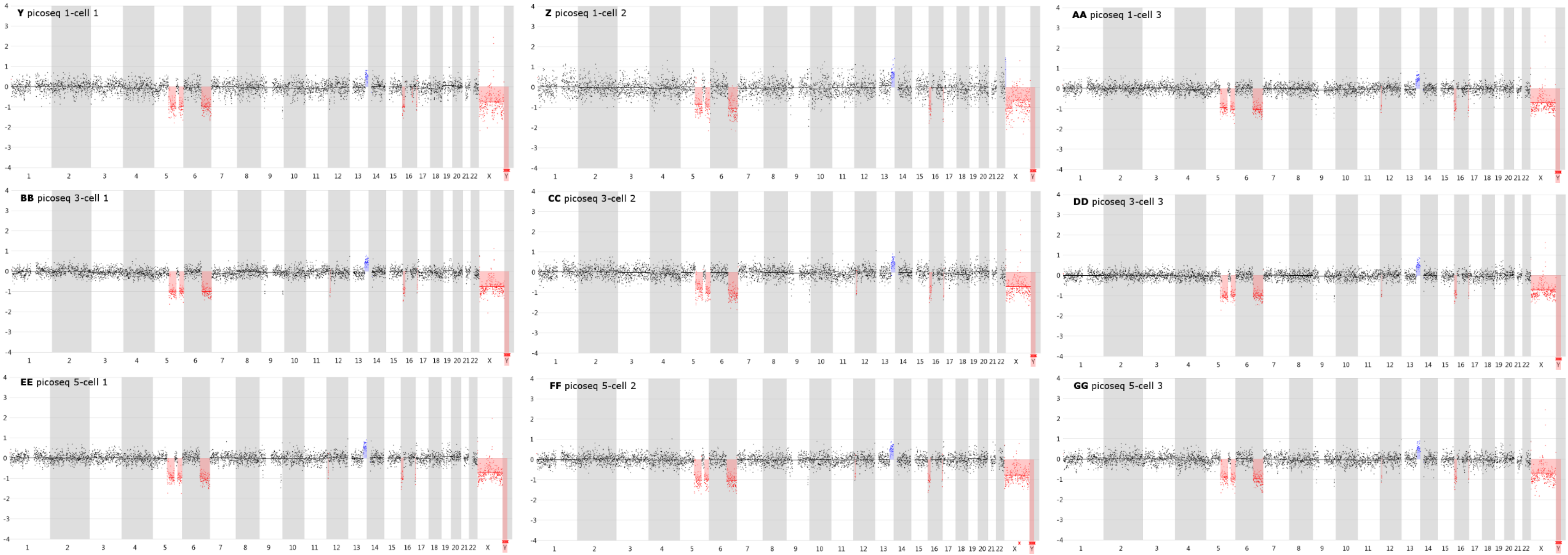

Supplementary Table S3: True and false positives called by Vivar using 500Kb windows. From left to right, separated by a black line: all true positives that are larger than 3Mb; true positives smaller than 3Mb that are called in at least 1 sample; false positives. The column headers indicate the location of the insertion (**in bold**) or the deletion. Two CNVs located on the same chromosomal arm are distinguished by subscript (1) or (2).

| | | Chr5q(1) | Chr5q(2) | Chr6q | Chr12p | **Chr13q** | Chr16p | Chr16q(2) | X-Chr | Chr9p | Chr9q | Chr16q(1) | Chr1p | **Chr1q** | Chr2p | Chr2q |
|---|---|---|---|---|---|---|---|---|---|---|---|---|---|---|---|---|
| Bulk | | √ | √ | √ | | √ | √ | √ | √ | | | | | | | |
| Ampli-1 | 1c1 | √ | √ | √ | √ | | √ | √ | √ | | | | | | | |
| | 1c2 | √ | √ | √ | √ | | √ | √ | √ | | | √ | | | | |
| | 1c3 | √ | √ | √ | √ | √ | √ | √ | √ | | | √ | | | | |
| | 3c1 | √ | √ | √ | √ | √ | √ | √ | √ | | | √ | | | | |
| | 3c2 | √ | √ | √ | | √ | √ | √ | √ | | | | | | | |
| | 3c3 | √ | √ | √ | | √ | √ | √ | √ | | | | | | | |
| | 5c1 | √ | √ | √ | | √ | √ | √ | √ | | | | | | | |
| | 5c2 | √ | √ | √ | | √ | √ | √ | √ | | | √ | | | | |
| REPLI-g | 1c1 | √ | √ | √ | | √ | √ | √ | √ | | | | | | | |
| | 1c2 | √ | √ | √ | | √ | √ | √ | √ | | | | | | | |
| | 1c3 | √ | √ | √ | | √ | √ | | √ | | | | √ | | | |
| | 3c1 | √ | √ | √ | | √ | √ | √ | √ | | √ | | | | √ | √ |
| | 3c2 | √ | √ | √ | | | √ | √ | √ | | √ | | | | | |
| | 3c3 | √ | √ | √ | √ | √ | √ | √ | √ | | √ | | | | | |
| | 5c1 | √ | √ | √ | √ | √ | √ | √ | √ | | | | | | | |
| | 5c2 | √ | √ | √ | √ | √ | √ | √ | √ | | √ | | | | | |
| | 5c3 | √ | √ | √ | √ | √ | √ | √ | √ | √ | √ | | | | | |
| DOPlify | 1c2 | √ | √ | √ | | √ | √ | √ | √ | | | | | | | |
| | 1c3 | √ | √ | √ | √ | √ | √ | √ | √ | | | | | | | |
| | 3c1 | √ | √ | √ | | √ | √ | √ | √ | | | | | | | |
| | 3c2 | √ | √ | √ | √ | √ | √ | √ | √ | | √ | | | √ | | |
| | 3c3 | √ | √ | √ | √ | √ | √ | √ | √ | | | | | | | |
| | 5c1 | √ | √ | √ | √ | √ | √ | √ | √ | | | | | | | |
| | 5c3 | √ | √ | √ | √ | √ | √ | √ | √ | | √ | | | | | |
| Picoseq | 1c1 | √ | √ | √ | | √ | √ | √ | √ | | | √ | | | | |
| | 1c2 | √ | √ | √ | | √ | √ | √ | √ | | | | | | | |
| | 1c3 | √ | √ | √ | √ | √ | √ | √ | √ | | | | | | | |
| | 3c1 | √ | √ | √ | √ | √ | √ | √ | √ | | | | | | | |
| | 3c2 | √ | √ | √ | √ | √ | √ | √ | √ | | | | | | | |
| | 3c3 | √ | √ | √ | √ | √ | √ | √ | √ | | | | | | | |
| | 5c1 | √ | √ | √ | √ | √ | √ | √ | √ | | | | | | | |
| | 5c2 | √ | √ | √ | √ | √ | √ | √ | √ | | | | | | | |
| | 5c3 | √ | √ | √ | √ | √ | √ | √ | √ | | | | | | | |

| | | Chr3p | Chr3pq | **Chr3q** | Chr3q | Chr4p | Chr4q$_{(1)}$ | Chr5p$_{(1)}$ | Chr5p$_{(2)}$ | Chr8p | Chr9q$_{(2)}$ | Chr10q$_{(1)}$ | Chr10q$_{(2)}$ | Chr12pq | Chr12q$_{(1)}$ |
|---|---|---|---|---|---|---|---|---|---|---|---|---|---|---|---|
| Ampli-1 | 1c1 | | | | | | | | | | | | | | |
| | 1c2 | | | | | | | | | | | | | | |
| | 1c3 | | | | | | | | | | | | | | |
| | 3c1 | | | | | | | | | √ | | | | √ | √ |
| | 3c2 | | | | | | | | | | | | | | |
| | 3c3 | | | | | | | | | | | | | | |
| | 5c1 | | | | | | | | | | | | | | |
| | 5c2 | | | √ | √ | | | √ | | | | | | | |
| REPLI-g | 1c1 | | | | | | | | | | | | | | |
| | 1c2 | | | | | √ | √ | | | | √ | √ | | | |
| | 1c3 | | | | | | | | | | √ | | | | |
| | 3c1 | | | | | | | √ | | | | | √ | | |
| | 3c2 | √ | | | | | | | √ | | | | | | |
| | 3c3 | | √ | | | | | | | | | | | | |
| | 5c1 | | | | | | | √ | | | | | √ | | |
| | 5c2 | | | | | | | √ | | | | | √ | | |
| | 5c3 | | | | | | | √ | | | | | | | |
| DOPlify | 1c2 | | | | | | | | | | | | | | |
| | 1c3 | | | | | | | | | | | | | | |
| | 3c1 | | | | | | | | | | | | | | |
| | 3c2 | | | | | | | | | | | | | | |
| | 3c3 | | | | | | | | | | | | | | |
| | 5c1 | | | | | | | | | | | | | | |
| | 5c3 | | | | | | | | | | | | | | |
| Picoseq | 1c1 | | | | | | | | | | | | | | |
| | 1c2 | | | | | | | | | | | | | | |
| | 1c3 | | | | | | | | | | | | | | |
| | 3c1 | | | | | | | | | | | | | | |
| | 3c2 | | | | | | | | | | | | | | |
| | 3c3 | | | | | | | | | | | | | | |
| | 5c1 | | | | | | | | | | | | | | |
| | 5c2 | | | | | | | | | | | | | | |
| | 5c3 | | | | | | | | | | | | | | |

| | | Chr12q$_{(2)}$ | Chr13q$_{(1)}$ | Chr13q$_{(2)}$ | Chr17q | Chr18q$_{(1)}$ | Chr18q$_{(2)}$ |
|---|---|---|---|---|---|---|---|
| Ampli-1 | 1c1 | | | | | | |
| | 1c2 | | | | | | |
| | 1c3 | | | | | | |
| | 3c1 | | | | | | |
| | 3c2 | | | | √ | | |
| | 3c3 | | | | | | |
| | 5c1 | | | | | | |
| | 5c2 | | | | | | |
| REPLI-g | 1c1 | | | | | | |
| | 1c2 | √ | | √ | | | |
| | 1c3 | | | | | | |
| | 3c1 | | | | | √ | |
| | 3c2 | | | | | √ | |
| | 3c3 | | √ | | | √ | |
| | 5c1 | | | | | | √ |
| | 5c2 | | | | | | √ |
| | 5c3 | | | | | | |
| DOPlify | 1c2 | | | | | | |
| | 1c3 | | | | | | |
| | 3c1 | | | | | | |
| | 3c2 | | | | | | |
| | 3c3 | | | | | | |
| | 5c1 | | | | | | |
| | 5c3 | | | | | | |
| Picoseq | 1c1 | | | | | | |
| | 1c2 | | | | | | |
| | 1c3 | | | | | | |
| | 3c1 | | | | | | |
| | 3c2 | | | | | | |
| | 3c3 | | | | | | |
| | 5c1 | | | | | | |
| | 5c2 | | | | | | |
| | 5c3 | | | | | | |
